# Challenges of Implementing an Individual Randomized Controlled Trial (Women First: Preconception Maternal Nutrition Study) in a Rural Study Site: A Case Study From Pakistan

**DOI:** 10.1177/1178638819852059

**Published:** 2019-07-04

**Authors:** Sumera Aziz Ali, Umber Khan, Farina Abrejo, Sarah Saleem, Michael K Hambidge, Nancy F Krebs, Jamie E Westcott, Robert L Goldenberg, Elizabeth M McClure, Omrana Pasha

**Affiliations:** 1 Department of Community Health Sciences, The Aga Khan University, Karachi, Pakistan; 2 Department of Pediatrics, Section of Nutrition, University of Colorado Anschutz Medical Campus, Aurora, CO, USA; 3 Department of Obstetrics and Gynecology, Columbia University, New York, NY, USA; 4 RTI International, Durham, NC, USA; 5 Department of Population, Family and Reproductive Health, Johns Hopkins Bloomberg School of Public Health, Baltimore, MD, USA

**Keywords:** challenges, strategies, rural Pakistan, case study

## Abstract

Persistent global disparities in maternal and neonatal outcomes associated with poor maternal nutrition provided the genesis of the Women First (WF) study, an individually randomized controlled trial on preconceptional maternal nutrition. This article describes the challenges that arose in implementing this trial related to nutrition or diet of the mother, in District Thatta-Pakistan. During different phases of the study, we encountered problems in identifying the eligible participants, taking consent from couples, randomizing participants in different arms, conducting biweekly follow-up visits on time, ensuring compliance to the intervention, and measuring the primary outcome within the 24 hours of birth. Each challenge was itself an opportunity for the research team to address the same through effective coordination and teamwork. Moreover, with adequate resources and dedicated staff with diverse backgrounds, it was possible to implement the WF study across the widely scattered geographic clusters of District Thatta. In addition, there are some broad strategies that could be applied to other studies such as very close contact either in person or at least by talking to mothers via phones and rapport with the study participants, the study leadership of country coordinator and the field supervisors to build trust between those on front lines and the study leadership. Moreover, continuous monitoring and supervision with frequent training and refreshers were also found to be more important to assure the data quality and to meet the study targets. Community meetings were also found to be very helpful and effective to follow the participants for a long time. Researchers conducting a similar type of studies particularly in rural areas can learn many lessons from such experiences. Thus, the process of implementing the study in one of the rural areas of Pakistan provides an insight into where and how similar individual randomized trials might be deployed.

## Highlights

We encountered major challenges in implementing an individual randomized trial in the rural site of Pakistan. These included age estimation, taking consent from couples, conducting biweekly visits, maintaining cold chain for biological samples, ensuring compliance to the intervention and a variety of other field-related issues. We propose to ensure very close contact and rapport with the study participants, rigorous training at various steps, community meetings at different intervals, and teamwork to implement similar individual randomized trials in the remote areas of developing countries.

## Background of Women First study

The Women First (WF) study was an individually randomized controlled trial which means that every individual woman rather than a group of women was separately approached and randomized in the village by home visitor research assistant (HVRA). The HVRA assessed woman on some eligibility criteria such as age, hemoglobin (Hb) level, plan of delivery, pregnancy status, usage of contraceptives and allergies to groundnuts. Those women who were between 16 and 35 years old, had Hb levels of at least 8 g/dL, were not using any contraceptive methods, were not currently pregnant, wanted to deliver at facilities and were not allergic were included in the study. The primary aim was to determine the benefits to the offspring of women in poor, food-insecure environments of commencing a daily comprehensive maternal nutrition supplement  ⩾ 3 months prior to conception versus the benefits of commencing the same supplement at 12 to 14  weeks of gestation and also to compare offspring outcomes with those of a third trial arm who received no supplementation.^1^ Study’s intervention included daily 20 g lipid-based (118 kcal) multi-micronutrient (MMN) supplement and, for women who were underweight or had slow gestational weight gain, an additional protein energy supplement was provided. The primary hypothesis of the study was that in women living in poor, food insecure populations, commencing a maternal nutrition supplement at least 3 months prior to pregnancy (Arm 1) would result in significantly greater fetal linear growth than starting the same nutrition supplement at 12 to 14  weeks gestation (Arm 2) or than not providing this supplement (Arm 3).^1^ The details of the Women First study including design, study sites, follow-ups, various stages, and ethical approvals are discussed in depth elsewhere.^1^

Briefly, the Women First study used the existing research infrastructure of the Global Network for Women’s and Children’s Health Research sites in the Pakistan, India, Democratic Republic of Congo (DRC), and Guatemala. Central to each site was the development and maintenance of the Global Network Maternal Newborn Health Registry (MNHR) in defined geographic areas or clusters. Within these clusters, the Registry documented all pregnancies and their outcomes to 6 weeks post-delivery, providing population-based rates of stillbirth, maternal and neonatal mortality and morbidity, and health-care utilization.^2,3^

## Study Site and Research Team in Pakistan

In Pakistan, we implemented the WF study in District Thatta which is predominantly rural district bordering the two largest cities in the province of Sindh, Hyderabad, and Karachi. Thatta is located in the southernmost part of Sindh, 98 km east of Karachi.^4^ Despite its close proximity to these urban centers, Thatta was ranked 64th of 91 districts in the country on the Human Development Index.^5^ More recent reports show that Thatta has the lowest educational attainment score in the province and is ranked among the five lowest in the country.^6^ On the other hand, Thatta does have a large number of health-care providers spread throughout the district, and the proportion of women delivering at healthcare facilities is higher than the national average.^7^

**Figure 1.**
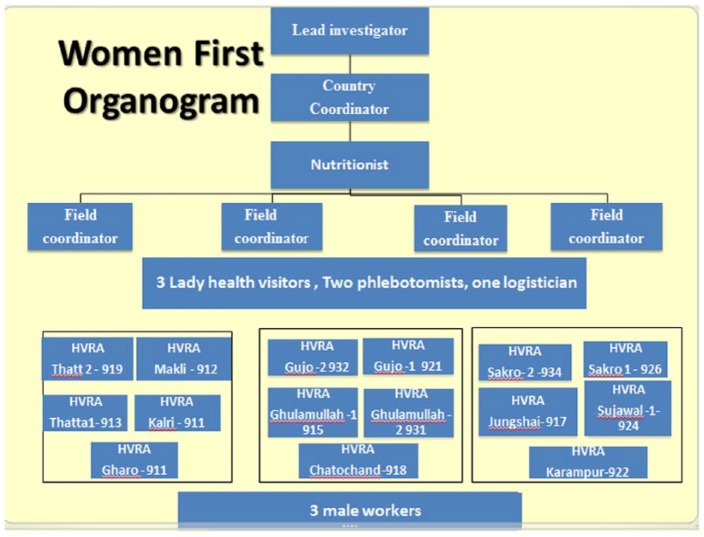
Organogram of Women First study in District Thatta Pakistan.

**Figure 2.**
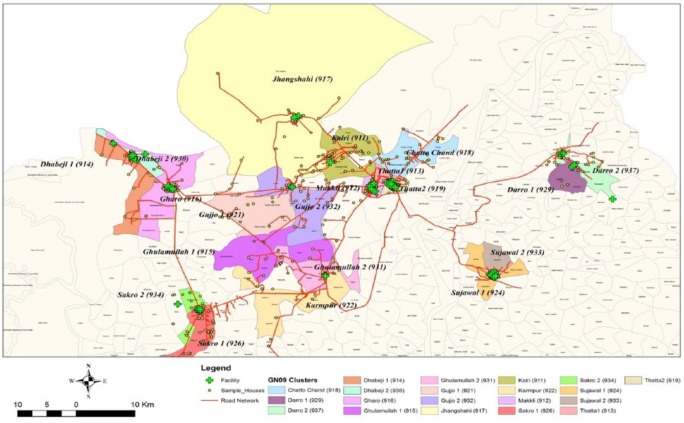
Geographic distribution of WF study sites in District Thatta Pakistan.

The WF study in District Thatta was conducted with the help of a diverse research team led by the principal investigator (PI). The team comprised the lead investigator, country coordinator, nutritionist, phlebotomists, a logistician, field supervisors, HVRAs, male workers, and support staff of data management system (DMS). The organogram of the study team is shown in Figure 1. The HVRAs and male workers primarily worked at the ground level under the supervision of the field supervisors and country coordinator, who helped them in making their field plans and also trained them on various domains of the study. One of the remarkable aspects of our team was the relatively low education level of the HVRAs, which contributed to the team leaders’ challenges and made the training even more critical. In order to complete the study effectively and timely, the research team had to coordinate not only with community leaders, and traditional birth attendants (TBAs) but had to build a strong rapport with the health care providers working at various primary and secondary level care facilities across the wide geographic area as shown in Figure 2. Each HVRA was assigned a cluster with a unique ID (eg, Gujo 1- 921) where they had to visit to enroll the women. Furthermore, HVRAs were recruited from the local communities, so they knew the traditions and the families very well and helped them to carry out activities smoothly.

## Brief Profile of the Study Participants

We screened around 3572 women in District Thatta, of those 2015 women were found to be eligible for certain criteria.^8^ These 2015 women also consented to participate in the study and were randomized to three Arms. The mean (SD) age, parity, and body mass index of the participants were 23.6 (4.2) years, 1.6 (1.6), and 19.9 (3) kg/m^2^, respectively. Around 81.6% of the women did not have formal education. With respect to various indicators of socioeconomic status, 63.7% of the households were found to have electricity, 33% of the households have their own flush toilet, 86.2% of the households have access to improved water, and only 12.1% of the households reported to use improved cooking fuel.^8^

## Protocol and Planning Phase of the Study

This study was a multi-country study being conducted in India, DRC, Guatemala, and Pakistan. The primary hypothesis of this study was that in women living in poor, food insecure populations, commencing a maternal nutrition supplement at least 3 months prior to pregnancy (Arm 1) will result in significantly greater fetal linear growth as determined by newborn LAZ than starting the same nutrition supplement at 12 to 14 week gestation (Arm 2) or than not providing this supplement (Arm 3).^1^

**Figure 3.**
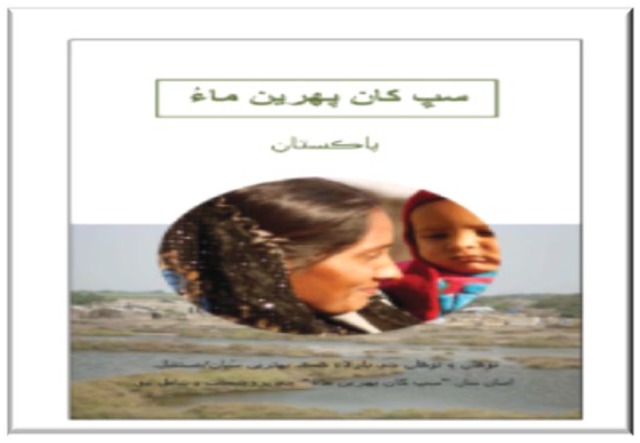
Flyer, distributed in the communities at the inception of the trial.

Overall for Pakistan, preparatory measures included the formation of a team consisting of members such as PI and Co-PI nutritionist, HVRAs, male workers, phlebotomist, and logistician. The main role of PI and Co-PI was to train the team members; conduct meetings with key stakeholders such as government, other non-governmental organizations, and communities to orient them about the project and to supervise, and monitor the activities of the trail. As an initial phase, PI and Co-PI got trained at UCD on protocol and manual of operations of the trial followed by training of HVRAs in Pakistan. Series of trainings were conducted on community mobilization, biweekly visits, data collection forms, anthropometric measurements of the woman, dietary recalls, food composition table, and biological sample collection and transportation by maintaining cold chain. A common manual of operations and data collection tools was developed by UCD and regional triangulate institute (RTI) and translated in the local language (Sindhi) of district Thatta Sindh Pakistan. In addition to translation, flyers were made in the local language to be distributed in the orientation meetings conducted in the villages (Figure 3). HVRAs conducting orientation meetings in the communities to inform them about the new trail and to identify the newly married women in the village. Newly married couples were identified and registered by household visits, making contacts with TBAs of the village via phones and by word of mouth.

## Challenges of Implementing Women First study

Many of the challenges faced in the study and the strategies taken to meet them reflect the teamwork and the diverse experiences of the team members. Usually, authors have reported challenges regarding the correct design of the randomized controlled trail.^9^ For example, whether the design chosen was appropriate to the research question and objectives of the study.^10^ But there is dearth on literature discussing the pragmatic challenges being faced while implementing an individually randomized trial mainly in rural area. For instance, one of the studies conducted in the DRC had discussed the challenges of implementing antenatal ultrasound screening in a rural study site which are unique to DRC.^11^

Therefore, it is important to highlight some of the challenges faced by us in implementing the trail in rural Thatta Pakistan with unique proposed solutions. This would help other researchers to plan similar type of trails in rural areas.

Some of these challenges such as age estimation, ensuring compliance to the nutritional supplement, hesitation in pregnancy test and taking consent from couples were unique to this trail. Therefore, innovative and unique strategies were adapted to address these challenges such as effective coordination, hard work, and commitment of the team members under the strong supervision of lead investigator and country coordinator. Although lots of challenges were faced by the team, a few of the major ones with their remedial are highlighted below.

### Screening

#### Resistance for pregnancy testing

During the screening of participants, HVRA faced the challenge of conducting pregnancy testing (PT) to confirm the pregnancy of around 3572 women in the remote villages. HVRAs and field supervisors overcame this challenge by using their counseling skills and by having a good rapport with the women. In rural areas, women get pregnant very frequently without resuming their periods after their last delivery. This lactational amenorrhea not only affects the woman’s health but also the newborn baby’s health. HVRAs and field supervisors motivated women to undergo the PT by talking to their family members and by making them realize the importance of women’s and baby’s health. HVRAs told mothers that if women did not allow them to conduct a PT, they would not come to know about their next pregnancy which might be an obstacle for women for their good care and timely planning for unknown pregnancy. This way, women usually agreed and allowed HVRAs to conduct their PT on time, which helped to enroll eligible women.

#### *Severe anemia (hemoglobin* *<* *8 g/dL)*

One of the common reasons for ineligibility was a low hemoglobin level (<8g/dL) of the screened women, and it was also later confirmed by the data analysis of WF for Pakistan site. Around 15% of the women in WF study were found to be severely anemic (<8 g/dL), 41.6% of the women were moderately anemic (8-10.9 g/dL) and 18% of the women had mild anemia (11-11.9 g/dL). As per study inclusion criteria, we had to enroll women with hemoglobin levels of at least 8 g/dL and severe anemia (<8 g/dL) was a challenge for HVRAs to enroll the eligible women. HVRAs were trained to counsel the women about consuming iron-rich vegetables and fruits, locally available inexpensively or even grown in their own farms. Moreover, HVRAs referred the women to hospitals for further care if required. HVRAs monitored hemoglobin levels after 2 to 3 months to enroll these women provided their hemoglobin were in an acceptable range(>8 g/dL). After 2 to 3 months, it was found that about 4% of the women had hemoglobin within an acceptable range and these women were enrolled in the study.

#### Age estimation

Another common challenge was to estimate the age of the women in rural areas. Due to the high illiteracy rate in Thatta mainly among women, it is very difficult to have actual ages of the women. They do not remember their exact age in years and no public records were available to verify the age of mother. However, HVRAs overcame this challenge by using different strategies such as verifying age from National Identity Cards if available and most importantly asking sequential validated questions in the following order which have been used in other studies conducted in the same study area in the past.^12^

1. When did you have your first period (menstruation) in life?/Tell me the approximate age when you had your first periods in life?
2. After how many years of having first periods, did you get married?
3. Can you recall that after your marriage, how many years did you take to conceive?
4. What is the age of your first child or can you show me where is your first child?
5. Is your first child still having polio drops (this estimates that child is within 5 years)?

These questions helped HVRAs to estimate the age of the mother in order to enroll the women of required age in the trial. Thus, we were able to complete the enrollment of required number of women of desirable age.

#### Family planning programs in urban areas

Another common reason for ineligibility was the usage of contraceptives by women in a few of the urban areas. Since this was an individually randomized trial, HVRAs were asked to screen women particularly in rural areas where the access of family planning (FP) programs was limited and women wanted to become pregnant.

### Enrollment of participants

Like other countries, we were also given some targets to enroll around 2000 women in the district Thatta Pakistan to complete the enrollment in a timely manner. It was a big challenge to enroll both nulliparous and multiparous women due to widely scattered study sites in District Thatta (Figure 2). The research team had to make some innovative strategies such as taking help from the staff of the MNHR, who enroll pregnant women by making door-to-door visits and by collaborating with a network of TBAs and health care providers in rural and urban areas, respectively.^2^ Second, we got help from a household survey, conducted in 2014 in the catchment sites of the MNHR. Another important strategy was to note the dates of mass marriage ceremonies in any village through the TBAs followed by meetings with the newly wed couples to screen the women for randomization. Furthermore, HVRAs also made regular contacts with community people via telephonic conversations, door-to-door visits and by arranging community meetings.

### Consent from couples

In the WF study, both the husband and wife had to consent for enrollment and study participation. Although it was relatively easy to take consent from women at the home, a major challenge was to take consent from their husbands. Husbands were either found at work or at home in the evening hours. Furthermore, in the extended family system of rural areas, couples usually discuss the consent form with their elder family members or educated community people to weigh the risks and benefits of the study before participation.

In order to take the timely consent from husbands, a few strategies were adopted which varied from urban to rural areas. For example, we hired a male worker to make contact with husbands via phone calls before meeting with them in person. Moreover, husbands of women used to return from their workstations in the evening hours usually after 6:00 or 7:00 pm. In that case, male workers had to arrange a meeting with husbands after these hours; whenever the husbands were available. Sometimes, male workers had to meet with educated community people and other family members along with husbands and these meetings used to last for 1 to 2 hours before the husbands completely understood the purpose of the study. In addition to this, HVRAs and male workers were also trained to leave the consent form at home for some time (at least 1 week) which helped the couple and other family members to discuss the study among themselves before study participation. The workers also adapted simpler strategies in the local language to explain the purpose of the study, the concept of randomization particularly the importance of Arm 3. By adopting these strategies, the process of taking consent from men specifically got expedited and around 99% of the couples gave consent for the study. This also helped workers to maintain a long-lasting relationship with the couples and followed them for approximately 5 years.

### Randomization across three arms

In order to randomize women across different arms, unique Screening ID and cluster IDs were assigned to every woman who was approached by HVRAs in the study. HVRA used to send the above-mentioned IDs to their respective field supervisors followed by the final receipt of randomization ID (unique ID) from data management system (DMS) established in the Department of Community Health Sciences at Aga Khan University in Karachi, Pakistan.

Since every HVRA in the rural area had a mobile phone, this helped to build the whole mechanism on short message service (SMS). Due to unavailability of the mobile network in the far-flung areas of District Thatta, sometimes SMS could not be communicated on time to randomize women promptly in remote clusters. This gap was critical for study implementation particularly at the time of randomization and at the time of outcome measurement within 24 hours of the birth of a baby. This challenge was addressed by training DMS staff to make phone calls to the field supervisors who in turn used to confirm from HVRAs either through phone calls or by meeting them in person every day about the receipt of randomization IDs. Furthermore, daily log sheets were maintained at DMS as well as field level to verify the number of randomized women by the end of the day on a daily basis for each HVRA. In addition to this, every field supervisor had to send her summary of work to the country coordinator who in turn double checked the number of randomized women before final authentication.

### Biweekly follow-ups

During the trial, each enrolled woman had to be visited on a biweekly basis to collect the relevant data.^1^ There were multiple challenges in conducting those biweekly visits which can be divided into two broad categories as follows.

#### Scattered field sites and workload

Field sites for the study were scattered across one-third of the district and most of the geographic areas had no infrastructure such as paved roads which was a great challenge particularly during the rainy season. Another challenge was to cover a huge number of follow-up visits on a single day ranging from 15 to 40 follow-ups per day per HVRA.

A few strategies were adopted to address the above challenges such as the distribution of vehicles and staff was done according to the workload. The hiring of more staff was done to cover the follow-ups on time. On average, there were 115 women assigned to each HVRA and more staff was sent to the areas where a huge number of participants had to be covered and this was especially true for rural areas which were very far where HVRA either had to travel around 150 km per day or HVRA had more than 100 participants under her supervision. Moreover, two to three HVRAs used to move in one vehicle from the Central Office of District Thatta to reach the particular cluster, where HVRAs used to visit women on their feet in the single village having more follow-ups. Furthermore, a route wise follow-up plan for every village was made to save time and reduce the high transportation costs. An effective biweekly follow-up plan was made by the field teams to cover all follow-ups in the same village on the same day to save time and resources. Moreover, HVRAs were given sheets with names and IDs of participants to track which woman/participant was due for an individual HVRA and two different HVRAs’ provided cover to each other to meet their targets whenever needed. In this way, the workload was distributed among HVRAs’ according to village route after getting off from vehicle at a common point. During the rainy season, a special vehicle with the 4-wheel drive (vigo vehicle) was used in the muddy areas, where the use of our usual small van (hiroof) could not work.

#### Participant’s availability on the day of follow-up

Another challenge was the uncertainty about the availability of the study participant during bi-weekly visits. There were a few reasons for this, such as most of the enrolled women used to leave their homes in the early morning to support their husbands on the farms for their daily earnings. Second, it was a tradition for nulliparous women to visit their parents’ homes very often particularly during their first pregnancy. Third, along with household chores, women had to take their children to the healthcare facilities or spiritual healers at shrines due to the illness. Finally, around 10% of the study follow-ups had to be done outside the catchment area due to the relocation of study participants either due to floods, rainy seasons or permanent migration. Due to these challenges HVRAs’ had to visit the women at their new locations. either on farms or at the health facilities or even at shrines. In case of relocation, the field team had contact details from the neighbors which helped the team to cover the follow-ups on the new address of the participants. Furthermore, the team also used to make a revisit plan to cover the pending follow-up within a window period as per protocol.

### Calendar marking

In the study, participants had to mark calendars regularly.^1^ High illiteracy was a big challenge for women to mark their calendars properly. Second, women were not habitual of marking calendars and they also used to lose the pens or pencils provided to them for calendar marking.

In order to overcome the above challenges, regular counseling was done until the calendar was understood by study participants. Second, we identified an active person from the community, who could be either another active study participant or husband of the enrolled participant or a voluntary worker from the community who could help the participants to mark the calendars in the same village on the fixed time. Instead of using pens or pencils, we advised women to use the coal from their kitchens to mark the calendar. Reminders were given by field teams through phones to mark the calendars regularly until the biweekly visits were made.

### Compliance

Compliance to the intervention was one of the important goals of the study, and challenges included women’s complaints of strong smell/taste, side effects (nausea, abdominal fullness, loose motions) particularly during pregnancy. Another challenge was the consumption of sachets by the children of the study participants due to food insecurity issues in far-flung areas.

Community meetings were conducted, where women were counseled to consume the supplement every day by sensitizing them about the importance of consuming sachets for themselves and for their upcoming baby. Furthermore, women were told to consume the supplement along with certain foods such as by spreading it on chapatti, mixing with lassi (yogurt based drink) or tea or consuming supplements with sour foods such as raw mango. Women were also told to consume the sachets in small portions throughout the day rather than consuming it as a whole.

These suggestions mostly applied to women consuming only Supplement 1. Since there were more complaints about Supplement 1 as compared to Supplement 2, those women who consumed both Supplement 1 and Supplement 2 were happy due to the better taste of Supplement 2. Furthermore, we had also conducted meetings with health-care providers to orient them about the study and this was found to be helpful when participants consulted with health-care providers to participate in the study and to clear their misconceptions and skepticism about supplements if any. With these strategies, we were able to ensure the compliance with the intervention and interestingly we also got positive feedback from mothers to feel better after consuming supplement. Furthermore, children were given roasted rice to make sure that they should not consume the supplement and women were also told that the supplements are made only for them, not for their children. In addition to this, extra sachets were given to the enrolled women as a backup emergency supply during the public holidays, participant’s women and participant’s visit outside the area to ensure better compliance to the intervention.

### Hot weather

The temperature in District Thatta is often very high during the summer 48ºC (118ºF) and even during early winter. As per protocol, sachets had to be kept below 30ºC (86ºF), and various biological samples (stool samples, breast milk, buccal swabs, etc) which were taken at the homes had to be transported between 2ºC and 8ºC from the field to the local office in Thatta.^1^ Moreover, due to dust and hot weather, HemoCue machines in the field got affected, which was an obstacle to measure the hemoglobin in the villages.

In order to maintain the required temperature, the central office in Thatta was equipped with an air conditioner where the sachets were stored. On the way from office to homes of the participants, sachets were transported in Coleman boxes with ice packs. While at home, clay urns were provided and women were told to keep sachets inside those urns which had a layer of water at the bottom in a separate pot to make the sachets cool throughout the day. The field team was given temperature monitoring devices to ensure that the temperature of an urn is below 30ºC.

Likewise, biological samples were also transported in Coleman boxes from the field to the local office either with ice packs or dry ice depending upon the temperature. Log sheets of temperature monitoring were maintained and the temperature was monitored at various levels such as central office, during transportation and at homes. Similarly, HemoCue machines were transported in the field with proper wrapping in zip lock bags in Coleman boxes to avoid dust and overheating of devices.

### Outcome measurement within 24-hours of the birth

It was one of the biggest challenges to measure the outcome (birth length) of a baby within 24 hours of birth and to collect the placental samples on time. In rural areas, usually women do not plan for their delivery ahead and they move outside the catchment area to deliver their baby at their mother’s home or some other place or end up with home deliveries.

Since it was highly important to measure the primary outcome of study within a given time period, every member of the field team was vigilant 24/7. The field team was given a list of expected dates of delivery (EDD) based on the ultrasound findings, which helped to monitor the date of delivery vigilantly every day after 5 months of pregnancy. Since daily visits were not feasible for the HVRAs, they took information from TBAs, family members via phones on a daily basis unless they met women during bi-weekly visits. Furthermore, project leads also called HVRAs to get updates about each pregnant woman of their assigned cluster.

In order to collect the placental samples and to measure the birth length within 24 hours, women and their families were sensitized about institutional delivery. We provided phone numbers of field team members to women for calling us any time and awareness was raised among enrolled women about the availability of 24/7 free transportation to take them to the facility at the time of delivery. A separate team was hired at the facility level to make the necessary coordination with field supervisors and HVRAs to measure the outcome and to collect the placental samples on time.

Furthermore, necessary arrangements were made to measure the outcome of those babies whose mothers delivered outside the catchment areas by coordinating with families, field teams and the support staff managing the transportation for WF study. Sometimes, the field team had to leave very early in the morning to travel 400 km which was not possible without teamwork.

## Conclusion

Every community-based field trial has its own challenges with different field realities and recognition of which helps not only bring opportunities for learning but also help researchers to prepare for the future. The researchers conducting a similar type of studies particularly in rural areas can learn many lessons from such experiences.

Overall, given the above discussed challenges, some proposed broad strategies could be applied to other studies such as very close contact and rapport including both in person and by phone with the study participants, the study leadership of country coordinator and the field supervisors which was provided to HVRAs’ which was incredibly important to build trust between those on front lines and the leadership of the study. The close contact can be ensured cost-effectively by hiring a staff of the project from the same village who can be easily accessible to woman whenever needed or by making phone calls to remain in touch with participants by taking advantage of available call packages. Furthermore, in the rural areas, we have observed that women usually need initial rigorous and effective meetings to establish the rapport with workers which can last for many years even without regular in-person meetings.

Moreover, initial rigorous and frequent training and refreshers at various steps were also found to be more important to assure the quality of data and to meet the study targets. Community meetings at different intervals were also found to be very helpful and effective to follow the participants for a long time. Thus, the process of implementing the study in one of the rural areas of Pakistan provides an insight into where and how similar individual randomized trials might be deployed.

Some of the challenges such as taking consent from couples could have been avoided before implementing the study. For example, consent of male workers could have been taken easily by hiring a separate staff for night shift who could have easily accessed to husbands of women in the evening after they used to come from their work. Similarly, give the bitter taste of nutritional sachets, ways of consuming it by mixing with other food items of different weathers could have been identified apriori to ensure good compliance to the intervention. Finally, the schedule of training workshops with refreshers could be planned by assessing the needs of staff and dividing them into various categories such as those who are sharp and intelligent and can catch things very quickly, others are the ones who are bit slower to gain and need more refreshers with practical examples and role-plays in the field.

Therefore, it is recommended that researchers implementing a similar type of trial in rural areas should be vigilant and plan their activities effectively according to the existing cultural practices, norms, and needs of the community.
